# Nanopore sequencing of IPNV vp2 gene in Peruvian Andean trout (*Oncorhynchus mykiss*) cultures

**DOI:** 10.1128/mra.00190-24

**Published:** 2024-08-20

**Authors:** Stephanie Tapia, Joseph Orellana, Yerson Duran, Jose Rodriguez, Derly Angulo, Luz Dominguez-Mendoza, Sandra Grabiel, Jose Silva, Romina Caballero, Katherine Zapata, Muriel Gómez, Luis Tataje-Lavanda, Rodolfo Velazco

**Affiliations:** 1 Laboratorio de Sanidad Acuícola—Sede Callao. Organismo Nacional de Sanidad Acuícola (SANIPES), Lima, Peru; 2 Escuela Profesional de Medicina Humana, Universidad Privada San Juan Bautista, Lima, Peru; DOE Joint Genome Institute, Berkeley, California, USA

**Keywords:** nanopore, IPNV, vp2, amplicon, trout, *Oncorhynchus*, Peru

## Abstract

Nanopore sequencing of the infectious pancreatic necrosis virus (IPNV) vp2 gene from Andean trout cultures in Peru reveals genogroups 1 and 5. This insight aids in understanding strain diversity and pathogenicity, vital for effective disease surveillance, and control measures in aquaculture.

## ANNOUNCEMENT

Trout farming is vital for Peru’s Andean economy but faces a significant threat from infectious pancreatic necrosis in its cultures (1–4). The causative agent, infectious pancreatic necrotic virus (IPNV), belongs to the family Birnaviridae, genus Aquabirnavirus, has a non-enveloped, single icosahedral capsid around 60 nm in diameter, with a genome comprising two double-stranded RNA (dsRNA) segments: A (~3,100 bp) and B (~2,784 bp). Segment A encodes the polyprotein (pvp2-vp4-vp3, 106 kDa), while segment B encodes the RNA-dependent RNA polymerase, vp1 (5). Birnaviruses are classified into seven genogroups (numbers 1–7), discerned through the vp2 gene’s ORF segment A phylogenetic analysis (5, 6). This study reports amplicon vp2 Nanopore sequencing of IPNV genogroups 1 and 5 within trout cultures in Peru’s southern Andean region. These samples were selected for later detection of the vp2 gene using conventional PCR.

Samples were collected from rainbow trout alevin specimens with clinical signs compatible with IPNV in December 2021 in the Apurimac region, and during 2022 in Puno and Huancavelica regions. The organs collected were the liver, kidney, and spleen (2) by specimen and each sample consisted of a pool of five specimens. These samples were confirmed to IPNV (vp1 gene) with reverse transcription quantitative real-time PCR (RT-qPCR) and then selected for later genotyping with the vp2 gene (6). Amplicon sequencing of the IPNV vp2 gene was amplified by conventional PCR and sequenced using Oxford Nanopore Technologies (ONT, UK). RNA extraction from 20 mg tissue samples used the ReliaPrep RNA Tissue Miniprep System kit (Promega, USA). RNA quantification relied on a Qubit 4 fluorometer (Invitrogen, USA), while cDNA synthesis employed the RevertAid First Strand Kit (Thermo Scientific, USA) with random hexamers.

Amplification of a 1,180 bp fragment of the vp2 gene utilized Hot Start High-Fidelity 2× Master Mix (New England Biolabs, USA) with the A1F/A2R primers (6). PCR products underwent 1.5% agarose gel electrophoresis, purification with the NucleoTraPCR kit (Macherey-Nagel, Germany), and quantification using a Qubit fluorometer. Sequencing library preparation followed the Rapid Barcoding SQK-RBK004 Kit protocol recommended by ONT, with sequencing conducted on a MinION Mk1C (ONT) sequencing platform using an R9.4.1 flow cell (FLO-MIN106D) for 4 hours (936.63 k reads; 577.49 Mb of passed bases; average QScore: 11). Basecalling of HAC (High Accuracy) bases and demultiplexing were performed using Guppy Software (Guppy v5.1.13). Fastq files underwent processing using the Galaxy platform (7, 8) and the NanoPlot tool (Version 1.28.2) (9) to obtain read statistics. Reads underwent trimming with the Porechop tool (Version 0.2.4) (10) and were filtered for quality (qscore ≥ 8) and length (900–1,800 bp) using the Filtlong tool (version 0.2.1) (11). Processed reads aligned to an IPNV reference sequence (NC_001915.1) (12) using Minimap2 (Version 2.26) (13). Finally, a consensus sequence was generated using the Medaka consensus tool (Version 1.4.4) (14). Amino acid 217 (15) analysis employed the Geneious program, with sequence features detailed in Table 1.

**TABLE 1:** Nanopore data sequencing from vp2 gene of IPNV samples

No	Accession number/SRA	Genogroup	Geographic location	Consensus length	vp2 protein amino acid 217 position^*a*^	Mean quality score	Average depth	Estimated N50	Reads generated
1	OP894434 (SRR28760295)	1	Puno	1,162	A	11.3	1,525×	1,196.0	62,379
2	OP894435 (SRR28760294)	1	Puno	1,168	A	11.4	1,306×	968.0	55,715
3	OP894436 (SRR28760290)	1	Puno	1,167	A	11.5	2,529×	866.0	66,835
4	OP894437 (SRR28760289)	1	Puno	1,159	A	11.3	175×	1,395.0	46,558
5	OP894438 (SRR28760288)	1	Puno	1,165	A	11.4	1,092×	1,137.0	54,409
6	OP894439 (SRR28760287)	1	Puno	1,160	A	11.2	1,299×	1,128.0	89,962
7	OP894433 (SRR28760286)	1	Huancavelica	1,175	A	11.4	4,654×	977.0	56,558
8	ON953147 (SRR28760285)	5	Apurimac	1,172	N	11.4	3,430×	716.0	21,336
9	ON953148 (SRR28760284)	5	Apurimac	1,163	N	11.5	2,197×	767.0	10,523
10	ON953149 (SRR28760283)	5	Apurimac	1,172	N	11.4	4,942×	760.0	24,822
11	ON706362 (SRR28760293)	5	Apurimac	1,172	N	11.5	12,179×	730.0	64,489
12	ON953150 (SRR28760292)	5	Apurimac	1,180	N	11.0	3,805×	640.0	37,936
13	OP894432 (SRR28760291)	5	Puno	1,160	T	11.2	1,081×	1,175.0	51,537

^*a*^ Virulent vp2:P217T, moderate vp2:P217P, low vp2:P217P (16).

**Fig 1:**
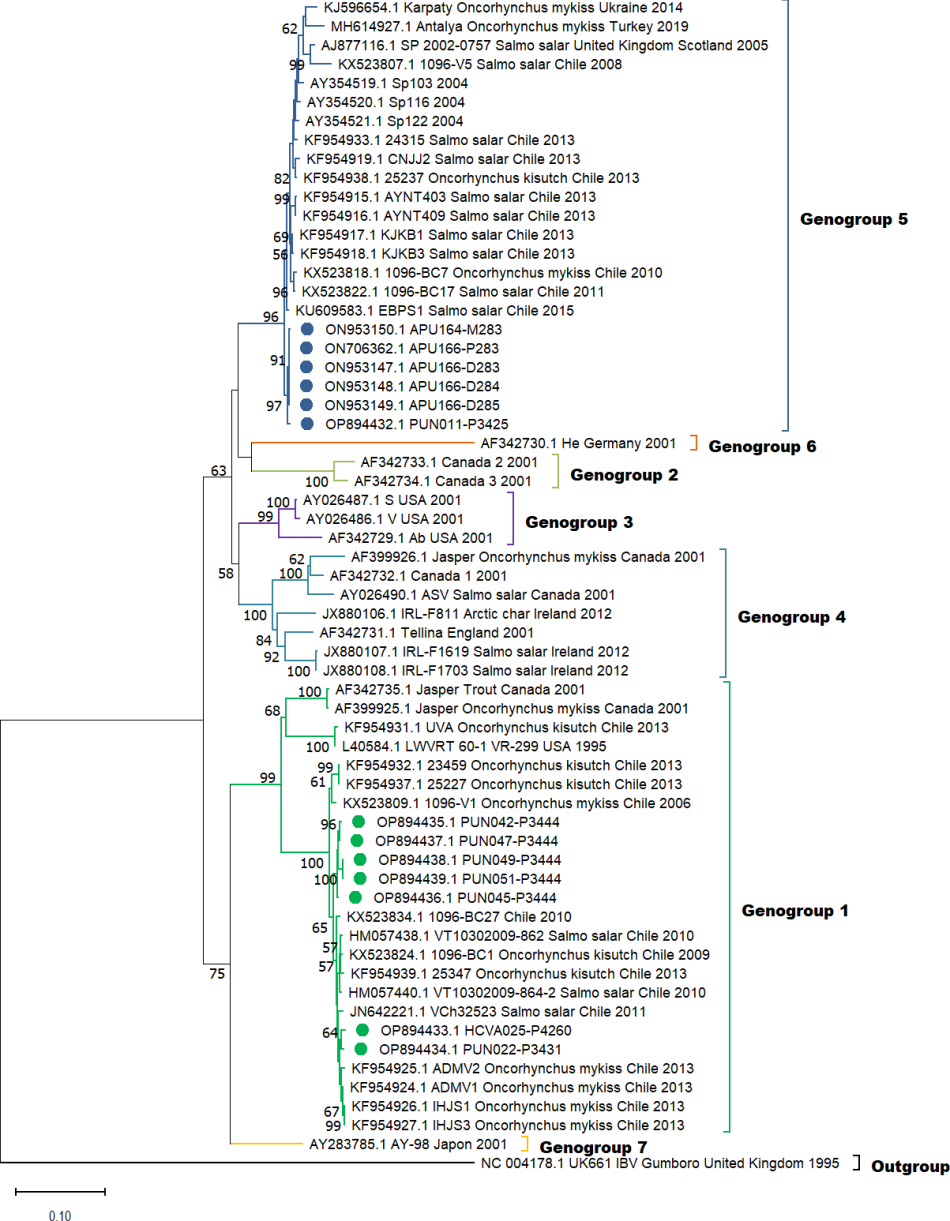
Phylogenetic tree based on nucleotide sequence comparisons of the vp2 gene, showing relationships between IPNV samples analyzed in this study (blue and green circles) and reference strains. Analysis was performed in the MEGA 11 program using the neighbor-joining method; confidence in tree construction was assessed using 1,000 bootstrap replicates. Bootstrap values greater than 50% are shown. The evolutionary distances were computed using the maximum composite likelihood method.

The phylogenetic analysis involved 13 sequences of the vp2 gene from Peruvian IPNV strains and 49 vp2 gene sequences of IPNV obtained from GenBank. These strains represent all IPNV genogroups (6, 16, 17). Multiple sequence alignments used the MUSCLE algorithm in MEGA 11 (18). Phylogenetic tree construction employed the neighbor-joining method in MEGA with 1,000 bootstrap replications, and substitution models were determined using the maximum composite likelihood method (Fig. 1). The samples evaluated belong to genogroups 1 and 5.

This study yields critical insights into the pathogenic strains endangering trout farming in Peru, facilitating disease surveillance and IPNV control through vaccine development.

## Data Availability

The vp2 gene consensus sequences have been deposited in GenBank under Accession numbers: ON953147, ON953148, ON953149, ON706362, ON953150, OP894432, OP894434, OP894435, OP894436, OP894437, OP894438, OP894439, OP894433. The Nanopore raw reads for this sequencing project (PRJNA1102916) are available under the following accession numbers: SRR28760295, SRR28760294, SRR28760290, SRR28760289, SRR28760288, SRR28760287, SRR28760286, SRR28760285, SRR28760284, SRR28760283, SRR28760293, SRR28760292, SRR28760291.
